# Cold Pressor Stress Cardiac Magnetic Resonance Myocardial Flow Reserve Is Not Useful for Detection of Coronary Endothelial Dysfunction in Women with Signs and Symptoms of Ischemia and No Obstructive CAD

**DOI:** 10.1371/journal.pone.0169818

**Published:** 2017-01-12

**Authors:** Sofy Landes, Sherwin Dela Cruz, Janet Wei, Ahmed AlBadri, Chrisandra Shufelt, Puja Mehta, Louise E. Thomson, Marcio A. Diniz, Xiao Zhang, John W. Petersen, R. David Anderson, Carl J. Pepine, Daniel S. Berman, C. Noel Bairey Merz

**Affiliations:** 1 Barbra Streisand Women’s Heart Center, Cedars-Sinai Heart Institute, Los Angeles, California, United States of America; 2 Samuel Oschin Comprehensive Cancer Institute, Los Angeles, California, United States of America; 3 Univerity of Florida, Gainesville, Florida, United States of America; University of Bologna, ITALY

## Abstract

**Background:**

Coronary endothelial function testing using acetylcholine is not routinely available, while non-pharmacological cold pressor testing (CPT) is considered an endothelial stressor. Noninvasive cardiac magnetic resonance imaging (CMRI) myocardial perfusion reserve index (MPRI) can detect coronary microvascular dysfunction (CMD). We evaluated if CPT stress CMRI MPRI could detect invasive coronary endothelial dysfunction.

**Methods:**

Coronary reactivity testing was performed in 189 women with symptoms and signs of ischemic but no obstructive coronary artery disease as previously described plus CPT stress. Subjects also underwent pharmacologic and CPT stress during CMRI (1.5 T). Statistical analysis comparing CPT MPRI between groups was performed by Welch`s t-test and Mann-Whitney where appropriate. Anderson-Darling test and Levene test were considered to verify the normality and homogeneity of variances assumptions. Correlation analyses between CPT MPRI and both invasive and noninvasive measures of CMD were performed using Spearman correlation.

**Results:**

While CPT MPRI correlated with pharmacological stress MPRI, it did not correlate with invasive measures of CMD including invasively measured responses to intracoronary (IC) adenosine, IC acetylcholine, CPT, or IC nitroglycerin. Additionally CPT MPRI was not significantly different between subjects with normal compared to abnormal pharm stress MPRI or normal compared to abnormal invasive CMD parameters.

**Conclusion:**

Despite correlation with pharmacological stress MPRI, non-invasive CPT MPRI does not appear to be useful for detecting CMD in symptomatic women.

## Introduction

Women with symptoms and signs of ischemia and no obstructive coronary artery disease (CAD) by angiography frequently have coronary microvascular dysfunction (CMD)[1, 2], which carries an adverse prognosis for cardiovascular events including myocardial infarction (MI), stroke, heart failure, and sudden cardiac death compared to normal controls. [3–11] Treatment targeting endothelial dysfunction can reduce angina, coronary spasm, heart failure, and stroke. [12–15] It is therefore important to establish the diagnosis in order to provide appropriate medical management.

The gold standard for diagnosis of CMD is invasive coronary reactivity testing (CRT). [16] While CRT has been shown to be safe [16] it is time consuming and requires an experienced interventionist with advanced training to perform, and therefor is not routinely available. Studies have demonstrated that cardiac magnetic resonance imaging (CMRI) with myocardial perfusion imaging has been shown to be predictive of death, MI, hospitalization for worsening angina in women with CMD. [17] Myocardial perfusion reserve index (MPRI), a semi-quantitative measurement on CMRI, has shown promise for non-invasive detection of CMD. Pharmacologic vasodilator stress MPRI (adenosine or regadenoson) is reduced in women with angina and coronary endothelial dysfunction, and predicts presence of invasive CRT abnormality. [18]

Cold pressor testing (CPT) is a non-pharmacologic stressor [19] which has been shown to elicit the same endothelial dependent response in the coronary microvasculature. [19–21] We hypothesized that CPT stress MPRI could detect invasive coronary endothelial dysfunction.

## Methods

### Study subjects

We evaluated 189 women with signs and symptoms of myocardial ischemia (chest pain and abnormal routine stress testing) and no obstructive CAD (<50% epicardial coronary stenosis in all epicardial coronary arteries on clinically indicated coronary angiography), who were enrolled in the National Heart, Lung, and Blood Institute (NHLBI)-sponsored Women’s Ischemia Syndrome Evaluation- Coronary Vascular Dysfunction (WISE-CVD) (clinicaltrials.gov NCT00832702). Details of the WISE study design have been described elsewhere. [18, 22] The Institutional Review Boards at Cedars-Sinai Medical Center and University of Florida Medical Center approved the study, and all subjects gave written informed consent before study participation.

### CRT protocol

Left heart catheterization, quantitative coronary angiography, and coronary reactivity testing were performed according to previously published protocol. [16, 23] CPT was subsequently performed by placing an ice pack on either the hand and forearm (n = 100), or the forehead (n = 89), for two minutes. Coronary angiography was performed following the third dose of adenosine and after each subsequently administered vasoactive substance. Vessel diameter for evaluation of change in coronary diameter to intracoronary (IC) acetylcholine, CPT, and IC nitroglycerin was calculated 5 mm distal to the Doppler wire. Change in coronary blood flow in response to IC acetylcholine (ΔCBF) was calculated from average peak velocity and coronary cross section area. Data was analyzed by WISE core laboratory, who were blinded to the clinical data.

### CMRI with CPT protocol

A standardized CMRI protocol and equipment were used (1.5 T Magnetom Avanto; Siemens Healthcare, Erlangen, Germany) as previously published. [24, 25] First-pass contrast perfusion imaging was performed using gadolinium contrast of 0.05 mmol/L/kg (Gadodiamide; Omniscan, Amersham, Piscataway, NJ) infused at 4 mL/s, followed by 20 mL saline at 4 mL/s. Cold pressor stress utilized an ice pack wrapped around either the hand and forearm (n = 89) contralateral to the contrast injection, or the forehead (n = 100), for 2 minutes prior to first-pass perfusion imaging, and removed after completion of the first pass perfusion imaging data acquisition. Resting first-pass perfusion CMRI was acquired 10 minutes later. MPRI was measured as a ratio of the stress and rest upslopes of the whole myocardium, normalized to LV cavity blood pool input function, using CAAS MRV 3.3 software (Pie Medical Imaging, Netherlands), as previously described. [18]

### Statistical analysis

Baseline characteristics and clinical variables are presented as mean ± standard deviation (SD). All statistical analysis was performed using SAS (ver. 9.2; The SAS In1stitute, Cary, NC). Spearman correlations were used to evaluate for correlations between CPT MPRI and several measures of CMD. Welch`s t-test and Mann-Whitney where appropriate were used to evaluate for difference between those with normal and abnormal invasive CRT measures. Anderson-Darling test and Levene test were considered to verify the normality and homogeneity of variances assumptions. Normal coronary flow reserve in response to IC adenosine (CFR) was considered >2.5[9, 11], normal ΔCBF was considered ≥ 50% [5], normal change in coronary diameter in response to IC acetylcholine (ΔACH) was considered >0%[5], normal change in coronary diameter in response to CPT (ΔCOP) was considered >0%, and normal change in coronary diameter in response to nitroglycerin (ΔNTG) was considered >20%. [16] Statistical significance was considered p<0.05.

## Results

Patient baseline demographics as well as results of CRT and MRI are summarized in Table 1. The majority were Caucasian. Hypertension and history of smoking was common, while the frequency of dyslipidemia and diabetes was low. Of note the majority were overweight.

**Table 1:** Demographic and clinical variables.

Demographic	
Age, years (mean ± SD)	54 ± 11
Body Mass Index, kg/m^2^ (mean ± SD)	30 ± 8
Weight, kg (mean ± SD)	76 ± 19
Ethnicity (% Caucasian)	74
Current/former smokers (%)	46
Hypertension (%)	42
Dyslipidemia (%)	14
Diabetes Mellitus (%)	11
Heart rate, bpm (mean ± SD)	69 ± 11
Systolic blood pressure, mmHg (mean ± SD)	129 ± 21
Diastolic blood pressure, mmHg (mean ± SD)	62 ± 13
ACEi or ARB (%)	49%
Beta Blocker (%)	26%
Calcium Channel Blocker (%)	15%
Diuretic (%)	15%
Vasodilator (%)	3%
Aspirin (%)	68%
CFR (mean ± SD, (range))	2.7 ± 0.6 (1.3 to 5.4)
ΔCBF (%, mean ± SD, (range))	71 ± 87 (-68 to 456)
ΔACH (%, mean ± SD, (range))	0.5 ± 15 (-50 to 47)
ΔCOP (%, mean ± SD, (range))	3 ± 13 (-31 to 55)
ΔNTG (%, mean ± SD, (range))	15 ± 13 (-31 to 52)

ACEi = Angiotensin converting enzyme inhibitor, ΔACH = Change in coronary diameter in response to intracoronary infusion of acetylcholine, ARB = Angiotensin II receptor blocker, ΔCBF = Change in coronary blood flow in response to intracoronary infusion of acetylcholine, CFR = Coronary flow reserve in response to intracoronary infusion of adenosine, ΔNTG = Change in coronary diameter in response to intracoronary infusion of nitroglycerin, SD = standard deviation

**Fig 1:**
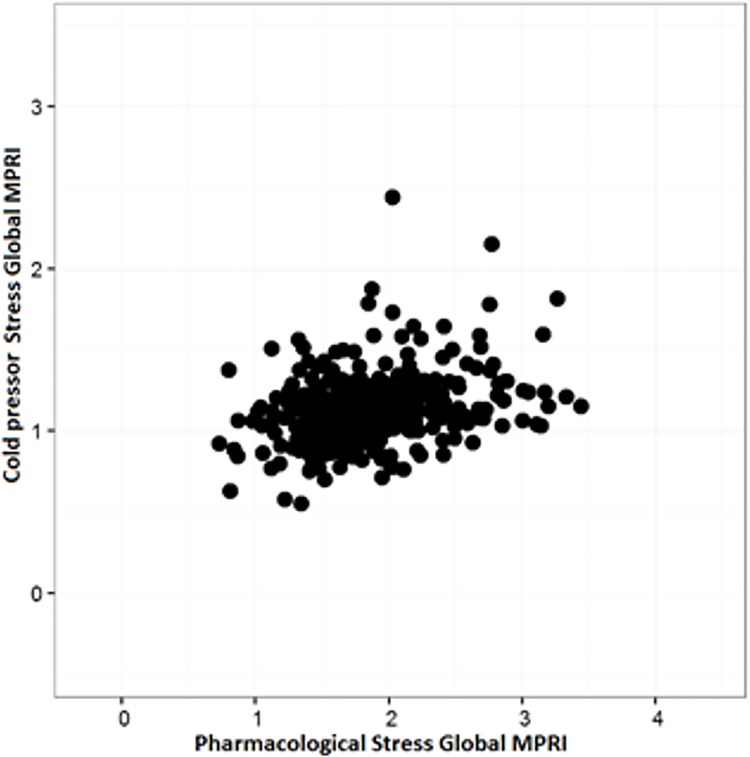
Correlation between CPT MPRI and pharmacological MPRI.

Mean CPT MPRI was 1.13 ± 0.22 (range 0.55–2.44). As demonstrated in Fig 1, correlation analysis showed a moderate positive correlation between global pharmacological stress MPRI and CPT MPRI. This was true when evaluating midventricular MPRI, subendocardial MPRI, and subepicardial MPRI. (Table 2) However there was no correlation between CPT MPRI and invasive CRT measures of CMD, including CFR, ΔCBF, ΔACH, ΔCOP, and ΔNTG.

**Table 2:** Spearman Correlation between pharmacological stress MPRI and CPT MPRI.

Variable	Correlation (CI 95%)	p-value
Global MPRI	0.32 (0.22; 0.41)	< 0.001
Midventricular MPRI	0.29 (0.18; 0.39)	< 0.001
Subendocardial MPRI	0.28 (0.18; 0.38)	< 0.001
Subepicardial MPRI	0.36 (0.26; 0.46)	< 0.001

The relationship between CPT MPRI and CRT was further examined by comparing the CPT MPRIs of subjects with normal and abnormal invasive CRT measures, as previously defined above. This analysis demonstrated that there was no difference in CPT MPRI between those with and without evidence of CMD, including pharmacological stress MPRI <1.8 vs pharmacological stress MPRI ≥1.8, CFR >2.5 vs. CFR ≤2.5, ΔCBF ≥50% vs ΔCBF <50%, ΔACH >0% vs. ΔACH≤0%, ΔCOP >0% vs. ΔCOP ≤0%, and ΔNTG ≥20% vs ΔCBF <20%. This remained true when comparing CPT midventricular MPRI, CPT subendocardial MPRI, and CPT subepicardial MPRI.

## Discussion

We assessed the utility of noninvasive, non-pharmacologic CPT during CMRI for the evaluation of coronary endothelial dysfunction in women with suspected CMD by comparing CPT CMRI results to pharmacological stress MPRI, and to invasive measures of CMD using traditional thresholds of normality. We found that despite a correlation between pharmacological stress MPRI and CPT MPRI, there was no difference in CPT MPRI between those with and without abnormal CMD pathways.

Vasodilator stress CMRI with semi-quantitative MPRI has been successfully evaluated for the non-invasive diagnosis of CMD, demonstrating in past studies that MPRI is lower in CMD cases compared to reference controls. [18] The mechanism of vasodilation by both regadenoson and adenosine is thought to be via stimulation of the adenosine A2A receptors on vascular smooth muscle cells which unlike acetylcholine represents a non-endothelial dependent pathway.[26, 27]

The cold pressor test originated in 1936, has been used to assess the function of the sympathetic branch of the cardiovascular system by observing the pressor response during immersion of one hand in cold water. [28] Nabel et al. showed that the normal response to CPT was vasodilation in normal and vasoconstriction in diseased coronary arteries, related to β-adrenoreceptor stimulation and possibly flow-mediated endothelial dilation or α2-adrenergic activity. [19, 20] Coronary diameter response to CPT is related to the capacity of the coronary microvasculature to dilate in response to intracoronary acetylcholine infusion [19–21], which is itself used to assess endothelial dependent micro- and macrovascular function by calculating ΔCBF and ΔACH. [16, 23] Multiple studies have shown that abnormal coronary response to CPT increases risk of adverse myocardial events. [10, 29]

To our knowledge no prior work has addressed the relationship between invasive pharmacologic testing of endothelial function and CPT CMRI. Endothelial function has been evaluated using CPT CMRI or CPT PET in other populations and shown to be reproducible [30]; however these were not compared to invasive measures. [31, 32] In contrast to our study which showed no relationship between CPT MPRI and invasive CMD measures, there is a single small study of ten patients by Meeder et al which showed that CPT stress non-invasive PET coronary blood flow increase is highly correlated with the intracoronary Doppler flow response to both ACh infusion and CPT. [33] There are several possible explanations for why this result differed from ours. To begin the aforementioned study was done in patients with obstructive CAD who were going for angioplasty, although the Doppler flow measurement was done in a non-stenotic vessel. Second, there is no currently established method to reproduce the changes discussed in this study—our CPT was done with an ice pack instead of using an ice bucket to completely submerge the hand as was done in the Meeder study. This was due to safety and feasibility constraints of the catheterization lab and MRI suite, and we cannot rule out the possibility of this affecting the CPT response. In our study CPT response was initially tested with an ice pack placed to the forehead (89/189 of the CRTs and 100/189 of the MRIs) instead of the forearm. In this cohort the systolic blood pressure and the heart rate responses were higher in those in whom CPT was done to the forehead. This difference may be due to in unintentional provocation of the diving reflex, which stimulates both the sympathetic and parasympathetic nervous system.[34, 35] Lastly response to CPT is heterogeneous, generally causing a relatively small increase in coronary flow compared to what is expected with pharmacological stress, and CMRI MPRI may not be sensitive enough to detect this smaller level of response.

Another possible explanation is that, as with all imaging, both CRT and MPRI show microvascular function at a single point in time- we did not record the time interval between the two studies (MRI and CRT) and whether patients had treatment in the intervening time period, which could improve the underlying dysfunction and alter results. Also, although a minority of patients had diabetes, it is possible that diabetic neuropathy could affect response to CPT.[36]

Our study has several limitations including those regarding heterogeneity of cold pressor testing techniques, physiologic response, and measurement of response. In addition, the WISE-CVD study is designed to evaluate symptomatic women, and therefor results cannot be applied to a more generalized population, including men and asymptomatic women.

## Conclusions and Implications

In women with signs and symptoms of ischemia and no obstructive CAD, CPT MPRI was not useful in the detection of coronary endothelial dysfunction. The identification of CMD is important because it carries an adverse prognosis [3–8] and directed treatment targeting endothelial dysfunction (e.g. external counterpulsation, HMG-CoA reductase inhibitors, ACEi) can improve the endothelial function. [12–15] For the benefit of the patient further investigation is needed to diagnose CMD non-invasively; however the gold standard for comprehensive assessment and diagnosis of CMD remains invasive CRT. [16, 37] Further work is needed to define normal ranges for CPT CMRI, as well as prognostic significance in women with suspected CMD.
